# Development of a *Dunaliella tertiolecta* Strain with Increased Zeaxanthin Content Using Random Mutagenesis

**DOI:** 10.3390/md15060189

**Published:** 2017-06-21

**Authors:** Minjae Kim, Junhak Ahn, Hancheol Jeon, EonSeon Jin

**Affiliations:** 1 Department of Life Science and Research Institute for Natural Sciences, Hanyang University, Seoul 04763, Korea; sciencekor89@gmail.com (M.K.); jkahn11@hanmail.net (J.A.); 2 Department of Genetic Resources Research, National Marine Biodiversity Institute of Korea, Seocheon 33662, Korea; hjeon@mabik.re.kr

**Keywords:** zeaxanthin, *Dunaliella tertiolecta*, EMS mutagenesis, salinity, light intensity, repeated fed-batch culture

## Abstract

Zeaxanthin is a xanthophyll pigment that is regarded as one of the best carotenoids for the prevention and treatment of degenerative diseases. In the worldwide natural products market, consumers prefer pigments that have been produced from biological sources. In this study, a *Dunaliella tertiolecta* strain that has 10–15% higher cellular zeaxanthin content than the parent strain (*zea1*), was obtained by random mutagenesis using ethyl methanesulfonate (EMS) as a mutagen. This mutant, *mp3*, was grown under various salinities and light intensities to optimize culture conditions for zeaxanthin production. The highest cellular zeaxanthin content was observed at 1.5 M NaCl and 65–85 μmol photons·m^−2^·s^−1^, and the highest daily zeaxanthin productivity was observed at 0.6 M NaCl and 140–160 μmol photons·m^−2^·s^−1^. The maximal yield of zeaxanthin from *mp3* in fed-batch culture was 8 mg·L^−1^, which was obtained at 0.6 M NaCl and 140–160 μmol photons·m^−2^·s^−1^. These results suggest that random mutagenesis with EMS is useful for generating *D. tertiolecta* strains with increased zeaxanthin content, and also suggest optimal culture conditions for the enhancement of biomass and zeaxanthin production by the zeaxanthin accumulating mutant strains.

## 1. Introduction

Zeaxanthin, a xanthophyll member of the carotenoids, is a yellow pigment that is produced by most photosynthetic organisms, including higher plants and many microalgae [1]. Zeaxanthin participates in cellular photo-protection by filtering, quenching, and/or scavenging excessive light energy [2]. Due to the strong antioxidant activity of zeaxanthin, it has been recommended for the prevention of cardiovascular disease, some types of cancer, and, especially, age-related macular degeneration [3,4].

Xanthophyll pigments, including zeaxanthin and lutein, have been used as food additives and food coloring agents, approved by the EU as E161h (zeaxanthin) and E161b (lutein). Market demand for xanthophyll pigments, mostly lutein, exceeds $250 million per year in the USA [5]. The zeaxanthin market is still young, even though the importance of zeaxanthin for macular healthcare has been consistently reported [4,6]. Despite the recommended daily xanthophyll intake of 6.0 mg [7], the xanthophyll pigment industry has suffered from low productivity. Marigold (*Tagetes erecta* L.) flower petals, which are the current major conventional source of xanthophylls, contain only ca. 0.03% (dw/dw) of xanthophylls [8]; thus, there is a demand for better sources for xanthophyll production.

Microalgae are emerging as major bio-resources for sustainable pigment production. In particular, some microalgal species have high lutein productivity; these include *Chlorella zofingiensis* [9], *Scenedesmus* sp. [10], and *Muriellopsis* sp. [11]. In earlier reports, zeaxanthin was isolated from two species of green algae [12,13] and a cyanobacterium [14]; these studies have described methods and efficiency of extraction and purification of zeaxanthin from respective wild type strains. The advantages of using microalgae for pigment production include (1) a higher growth rate and higher pigment content (about 1% of dry weight) in comparison with marigold flower petals and other higher plants, (2) daily harvesting under optimal operating conditions, and (3) a pigment extraction process that is shorter than that for marigold flowers [15,16]. However, small cell size, the presence of a rigid cell wall, and low cell density in cultures are hurdles for efficient pigment production. To achieve economically feasible pigment production, high pigment content and biomass productivity should be balanced.

In general, *Dunaliella* species are used to produce carotenoid pigments because they have two advantages. First, they accumulate carotenoids under high-salinity conditions, and thus do not compete with freshwater microalgae and land plants for potable water supply and arable land. Second, pigment extraction from *Dunaliella* is easier than that from other microalgae because it lacks a rigid cell wall [17]. The effects of salinity on carotenoid biosynthesis and the maximum density of microalgae were reported previously [18,19,20,21]. Cell division of microalgae tends to be increased in low saline conditions. Otherwise, carotenoids tend to be accumulated in high saline conditions. Therefore, determination of suitable saline growth conditions is vital for enhancing carotenoid production in *Dunaliella*.

The zeaxanthin-accumulating mutant of *Dunaliella tertiolecta* (*zea1*) was selected from a mutant library generated by ethyl methanesulfonate (EMS) treatment, and was investigated in terms of its responses to stressful conditions [22,23,24]. Since then, there have been no further studies regarding its zeaxanthin productivity, or further development or optimization of culture conditions.

In this study, we performed two different methods to achieve enhancement of zeaxanthin production; one was to increase cellular zeaxanthin content, and the other was to increase biomass and growth rate. Thus, we used random mutagenesis to generate mutants with increased zeaxanthin content, and attempted to optimize growing conditions for maximal zeaxanthin production. Several mutants with enhanced zeaxanthin accumulation were generated by EMS treatment, and the *mp3* mutant was selected as a promising candidate. We confirmed that salinity of the culture-medium, and light intensity, are crucial factors to control biomass and zeaxanthin content. We determined the optimum salinity of the culture medium, and optimum light conditions for zeaxanthin production, by the *mp3* strain. We also confirmed that zeaxanthin yield can be improved by repeated fed-batch cultivation under optimized culture conditions.

## 2. Results

### 2.1. Selection and Characterization of the Dunaliella Tertiolecta mp3 Strain

During the initial visual screening process, approximately 200 candidates were selected from about 8 × 10^5^ colonies obtained by chemical mutagenesis of the *zea1* strain with EMS on the basis of their yellowish-green coloration. After pigment profile analysis by HPLC, three mutants (*mut6*, *mut9*, and *mut12*) were selected on the basis of their relative zeaxanthin per chlorophyll *a* content (Figure S1). These mutants lacked neoxanthin, violaxanthin, and antheraxanthin, but accumulated considerable amounts of zeaxanthin, similar to the parental strain (*zea1*). From the preliminary results of zeaxanthin content and growth pattern, *mut12* had the highest zeaxanthin content and growth, and was therefore used in all further experiments. This *mut12* was renamed macular pigment mutant 3 (*mp3*) according to its pigment profile characteristics.

**Figure 1:**
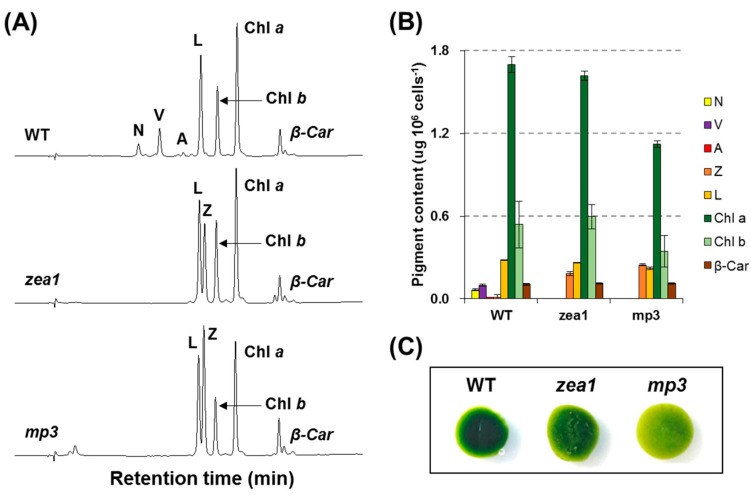
Pigment characteristics of wild type and mutant *D. tertiolecta* strains. (**A**) HPLC elution profile of total pigment extracts from the wild type, *zea1*, and, *mp3* mutant; (**B**) Pigment composition per 10^6^ cells (*n* = 3); (**C**) Colony color at the same cell density. N, neoxanthin; V, violaxanthin; A, antheraxanthin; Z, zeaxanthin; L, lutein; Chl a, chlorophyll a; Chl b, chlorophyll b; β-Car, β-carotene.

**Figure 2:**
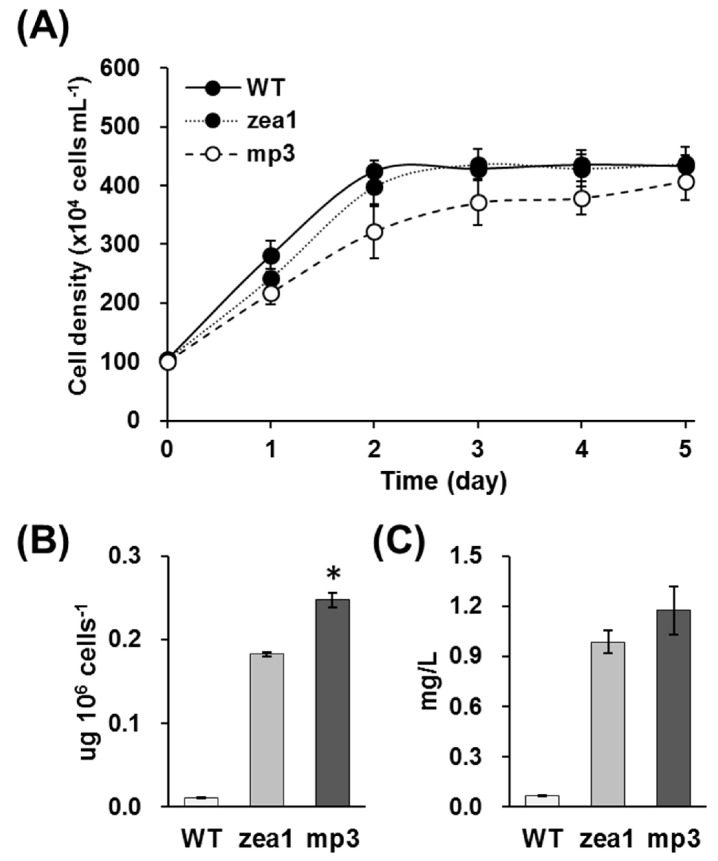
Comparative analysis of *D. tertiolecta* wild type, *zea1*, and *mp3*. (**A**) Growth patterns of three strains on 1.5 M NaCl medium; (**B**) Cellular zeaxanthin content of three strains (μg·10^6^·cells^−1^); (**C**) Zeaxanthin yield of three strains (mg·L^−1^). Statistical analyses were performed using Student’s *t*-test, * *p* < 0.05. All experiments were conducted in triplicate.

When grown as colonies on agar at 65–85 μmol photons·m^−2^·s^−1^, *mp3* appeared yellowish-green. The pigment profile was almost the same as that of the parental strain, but the number of chlorophyll molecules in *mp3* was greatly decreased (Figure 1); the *mp3* mutant had reduced total chlorophyll content and higher chlorophyll *a/b* ratio than *zea1*, and its autofluorescence signal was slightly decreased compared to *zea1* (Figure S2). *mp3* grew slower than other strains, and contained 10–15% more zeaxanthin per cell than *zea1* (Figure 2A,B). Zeaxanthin yield of *mp3* was about 19% higher than that of *zea1* under the same culture conditions (Figure 2C).

### 2.2. Effect of NaCl Concentration on Cell Density and Zeaxanthin Yield

**Figure 3:**
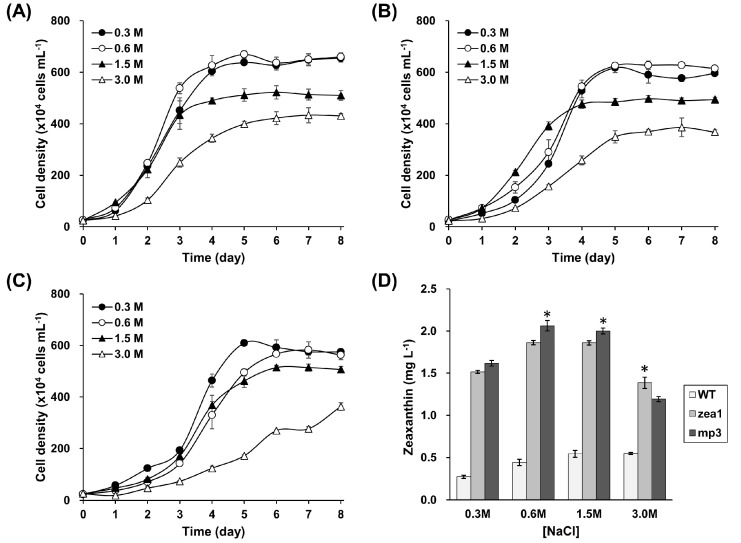
Effects of NaCl concentration on growth and zeaxanthin production in wild type *D. tertiolecta*, *zea1* and, *mp3* mutants. Growth pattern of the wild type (**A**), *zea1* (**B**), and *mp3* (**C**); (**D**) Zeaxanthin yield of wild type, *zea1*, and *mp3*. *Dunaliella* strains were cultured on media containing 0.3 M, 0.6 M, 1.5 M, and 3.0 M NaCl, respectively. Statistical analyses were performed using Student’s *t*-test, * *p* < 0.05. All experiments were conducted in triplicate.

Cell size was similar under different saline conditions; however, cell growth pattern, zeaxanthin content, and biomass were largely affected by salinity of the medium (Figure 3, Table S1). The maximal cell density at 0.3 M NaCl was similar to that at 0.6 M in the stationary phase (Figure 3A–C). However, the highest specific growth rate (μ) of the wild type reached 0.72 d^−1^ at 0.6 M NaCl; under the same conditions, the specific growth rate of *mp3* was 0.46 d^−1^. Specific growth rates were calculated during the exponential phase for the wild type (days 1–3) and *mp3* (days 3–6). Zeaxanthin yield seemed to be affected by salinity (Figure 3D). Table 1 shows the carotenoid content per 10^6^ cells at different salinities. In the wild type, low salinity-adapted cells tended to have high neoxanthin, violaxanthin, and antheraxanthin contents, but they tended to have low zeaxanthin content. Zeaxanthin content of both *zea1* and *mp3* mutants also tended to be reduced with decreasing salinity. The maximal cell density of all strains was observed at both 0.3 M and 0.6 M NaCl, but zeaxanthin content was highest at 3.0 M in the wild type, and at 1.5 M in the *zea1* and *mp3* mutants. Zeaxanthin yield (mg·L^−1^) of *mp3* was significantly higher than that of *zea1* at 0.6 M and 1.5 M NaCl (Figure 3). Zeaxanthin yield of *mp3* was 7%, 11%, and 8% higher than that of *zea1* at 0.3 M, 0.6 M, and 1.5 M NaCl, respectively, but was 14% lower at 3.0 M NaCl. Because the highest zeaxanthin yield of *mp3* occurred in a medium containing 0.6 M NaCl, we considered this medium as optimal for zeaxanthin production (Figure 3D).

### 2.3. Effect of Light Intensity on Growth Rate and Zeaxanthin Productivity

**Figure 4:**
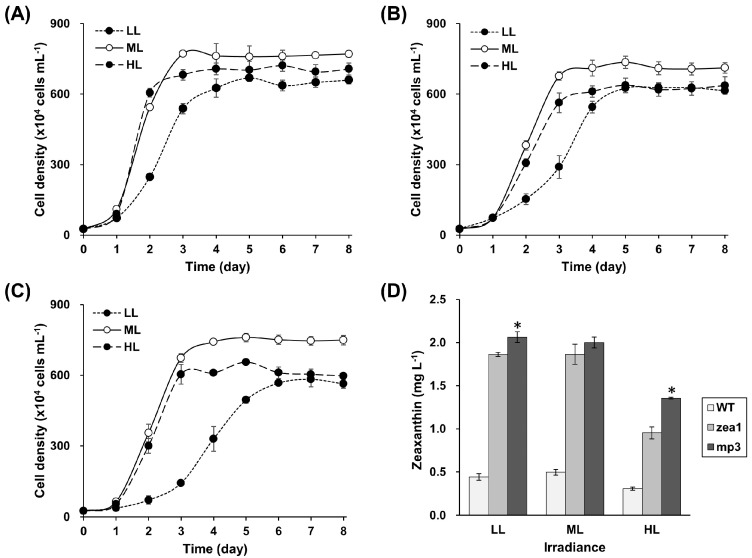
Effects of light intensity on growth and zeaxanthin production in wild type *D. tertiolecta*, and *zea1* and *mp3* mutants. Experimental light conditions were low-light (LL), mid-light (ML), and high-light (HL). Growth patterns of wild type (**A**), *zea1* (**B**), and *mp3* (**C**); (**D**) Zeaxanthin yield of wild type, *zea1*, and *mp3*. *Dunaliella* strains were cultured on media containing 0.6 M NaCl. Statistical analyses were performed using Student’s *t*-test, * *p* < 0.05. All experiments were conducted in more than triplicate.

Cell growth and zeaxanthin content in the wild type, and *zea1* and *mp3* mutants, were measured under three different light conditions. Increased irradiation promoted cell division in the wild type, *zea1*, and *mp3* (Figure 4). Interestingly, the growth rate of *mp3* was dramatically changed by light intensity (Table S2), increasing significantly from that at mid-light conditions. When the *mp3* mutant was grown under low-light conditions (65–85 μmol photons·m^−2^·s^−1^), its specific growth rate was only 0.46 d^−1^; however, it increased to 1.18 d^−1^ under mid-light conditions (140–160 μmol photons·m^−2^·s^−1^). Much stronger light (580–620 μmol photons) did not further affect the growth rate of *mp3* (1.21 d^−1^). This pattern was also observed in the wild type and *zea1*, as strong light did not affect the growth rate (0.97 d^−1^ and 1.11 d^−1^, respectively) in comparison with mid-light conditions. Specific growth rates were calculated during the same periods of the exponential phase for the wild type, *zea1*, and *mp3*. Carotenoid content is presented in Table 2. *Dunaliella* strains, investigated in this study, tended to have high pigment content under low-light conditions. However, zeaxanthin yield (mg·L^−1^) of the *mp3* and *zea1* strains under low- and mid-light conditions was higher than that under high-light conditions. Interestingly, *mp3* had a 42% higher zeaxanthin yield than *zea1* under high-light conditions (Figure 4D). Daily zeaxanthin productivity of *mp3* was 0.271–0.400 mg·L^−1^, which was 3–4 times higher than the productivity of the wild type under all light conditions.

### 2.4. Zeaxanthin Yield in Two Different Batch Culture Systems

**Figure 5:**
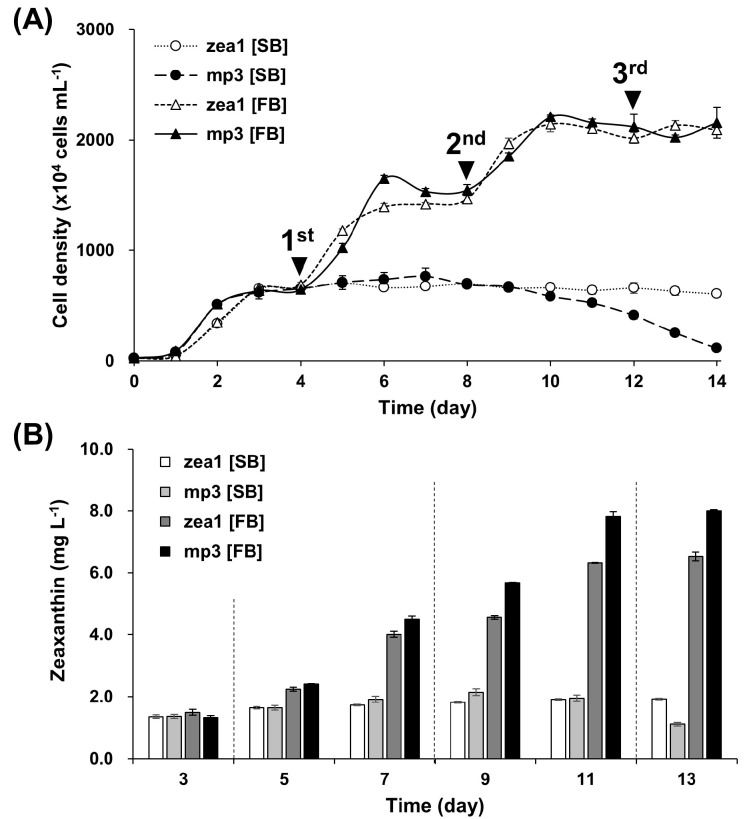
Comparison between single-batch (SB) and repeated fed-batch (FB) culture systems. Total replacement of the culture medium was conducted three times; on day 4 (1st), day 8 (2nd), and day 12 (3rd). (**A**) Growth patterns of *zea1* and *mp3*. Growth was improved at the previous two points but did not increase by more than 2500 × 10^4^ cells·mL^−1^; (**B**) Zeaxanthin yield of *zea1* and *mp3* according to two culture systems. The culture conditions are presented in detail in materials and methods.

To improve the yield of zeaxanthin and to determine its maximal yield, repeated fed-batch cultivation was performed in a 200 mL flask-culture system. Maximum zeaxanthin concentrations in repeated fed-batch cultures of *mp3* and *zea1* were 3.7 times and 3.4 times those in single-batch cultures, respectively (Figure 5B). This could be the result of the higher cell density observed in fed-batch cultures (Figure 5A). In detail, the maximum zeaxanthin yield of *mp3* was 8 mg·L^−1^, which was about 20% higher than that of *zea1* (6.5 mg·L^−1^). For a better comparison of zeaxanthin concentration between the two culture systems for the *mp3* strain, the amount of pigment per 10^6^ cells was calculated. Data analysis was performed with samples that had the highest zeaxanthin content during the experiment period. In fed-batch culture, it was 0.365 µg at 11 days after inoculation, which was about 15% higher than the content in single-batch culture (0.318 µg at 9 days), indicating that repeated fed-batch cultures may increase zeaxanthin yield and accumulation.

## 3. Discussion

Zeaxanthin has great potential as an antioxidant and is useful for macular healthcare. It is converted to antheraxanthin by zeaxanthin epoxidase in the xanthophyll cycle [25]. Hence, zeaxanthin is not accumulated in the cell to levels suitable for commercialization, except under stress. The use of genetically modified organisms (GMOs), which are generated by transformation to insert heterologous genes, for food applications is strictly regulated in the EU, the USA, and in the majority of other countries in the world [26]. Therefore, we developed a non-GMO strain and identified its optimal culture conditions to enhance zeaxanthin production. Our EMS mutant, *mp3*, accumulates zeaxanthin to higher levels (by 10–15% on per-cell basis) than the well-known mutant *zea1* (Figure 2B).

Random mutagenesis with chemical agents is probably the simplest method to genetically improve industrial microalgae. EMS is an alkylating agent that induces random mutations in genomes by nucleotide substitution, especially by guanine alkylation. This agent forms nucleotide adducts, causing them to form mispairs with their complementary bases [27,28]. Chemical mutagenesis is classified as a non-GMO method because it does not require the insertion of heterologous genes or vectors [29]. Including the *D. tertiolecta zea1* mutant, there have been many reports of EMS mutants with increased biomass and improved productivity of high-value products [30,31,32,33]. Although this is a powerful method for obtaining improved mutant lines, it is difficult to identify the mutations responsible for the mutant phenotypes. Only in a few random mutants have the mutated pathways related to phenotypes been suggested [22].

The *mp3* mutant had notable changes in physiological features. One of them is color. When we inoculated cells on solid culture plates at the same density, the *mp3* colonies were bright yellowish-green. The pale-color phenotype has been reported for several truncated antenna mutants, which have lower total chlorophyll content, higher chlorophyll *a/b* ratio, and a lower growth rate than the wild type under low-light conditions. There are several reports emphasizing the advantages of smaller-sized chlorophyll antenna mutants. The truncated, light-harvesting antennae are photosynthetically more productive under high-light and high-density culture conditions than cultures with fully pigmented cells. This improvement is due to alleviation of over-absorption, and dissipation of excess energy by the outer layer of cells in the culture, resulting in improved light penetration deeper into the culture and culture productivity [34,35]. Our results showed that *mp3* could be considered as an antenna mutant. *mp3* had reduced total chlorophyll content and a higher chlorophyll *a/b* ratio than the parental strain, *zea1*. Also, the specific growth rate of *mp3* was lower than that of the wild type and *zea1* under low-light conditions, but the specific growth rate of *mp3* was higher than those of wild type and *zea1* under mid- and high-light conditions (Table S2). In addition, our data showed that *mp3* has higher zeaxanthin productivity than *zea1* in high-light conditions (Figure S3). These results suggest that the *mp3* mutant could be more productive than the parental *zea1* strain under high-light conditions and higher cell density conditions. Although the *mp3* mutant has several significant phenotypes of antenna mutants, it is difficult to elucidate the exact genotype of EMS random mutants without whole-genome sequencing.

Manipulation of the β-carotene branches of the carotenogenic pathway, such as lycopene cyclase β (*LCYB*) and β-carotene hydroxylase (*CHYB*), could enhance the production of zeaxanthin precursors. The mutation in *mp3* may have affected these genes to enhance the production of zeaxanthin. In fact, overexpression of *Crocus sativus LcyB* in *Arabidopsis thaliana* resulted in accumulation of β-carotene and lutein [36], and overexpression of *A. thaliana ChyB* in *A. thaliana* resulted in accumulation of zeaxanthin [37]. Recently, a strategy has been reported to increase zeaxanthin content in *Chlamydomonas reinhardtii* by knocking out genes that encode the components of the carotenoid pathway (*ZEP*) and the signal molecules for antenna assembly (*FtsY*) [38]. This strategy uses the RNP-mediated CRISPR/CAS9 system, which is considered a non-GMO methodology. If the exact genotype of *mp3* is identified, this gene can be edited to increase zeaxanthin content in other microalgae.

Generally, large biomass and high growth rate are required to obtain high productivity; thus, there have been many attempts to enhance productivity by optimizing culture conditions [39]. There are several key factors in growth regulation, including salinity, temperature, pH, carbon source, and nutrients. Salinity is one of the most important environmental factors that affect physiological and biochemical responses in microalgae [40]. A number of studies have shown that the highest β-carotene content in *Dunaliella salina* is achieved under high-salinity conditions [19], and the increase in salinity increases cellular carotenoid content in wild type *D. tertiolecta* [18]. In our study, zeaxanthin tended to accumulate proportionally to a salinity increase in wild type *D. tertiolecta*. However, our results show that extremely high-salinity conditions are not optimal for increasing zeaxanthin production. On the basis of our results, we chose 0.6 M NaCl as the optimal salinity of the culture medium, which is similar to that of natural seawater (0.581 M); cultivation costs using natural seawater could be lower than those with artificial seawater. Also, the use of natural seawater could avoid competition with industries that use fresh water.

Although the maximal biomass of *mp3* was highest in 0.6 M NaCl media, additional optimization of environmental factors was necessary to increase its relatively low growth rate, thus, we varied light intensity. Light is an essential environmental factor for photosynthetic organisms. Adequate light energy is good for microalgal growth, but excessive energy has negative effects on the cell. Thus, excessive energy is dissipated as heat through a photoprotective mechanism—non-photochemical quenching [41]. In terms of the relationship between input (cost) and output (productivity), irradiation with excessive light would be wasteful. For this reason, there are many reports on optimization of light intensity to achieve the best conditions for microalgal growth rate. In most microalgae, photosynthesis becomes saturated at about 30% of total solar energy (1700 to 2000 μmol photons·m^−2^·s^−1^), and some picoplankton species grow at optimal rates at 50 μmol photons·m^−2^·s^−1^, and are photo-inhibited at 130 μmol photons·m^−2^·s^−1^ (reviewed in [42]). Tang et al. [43] reported that the growth rate of *D. tertiolecta* was increased with increasing light intensities from 100 μmol photons·m^−2^·s^−1^ to 350 μmol photons·m^−2^·s^−1^. Our results show a similar pattern: the growth rates of wild type, *zea1*, and *mp3* increased sharply with an increase in light intensity, and saturated at >140 μmol photons·m^−2^·s^−1^. We chose mid-light intensity (140–160 μmol photons·m^−2^·s^−1^) as the optimal light condition because excessive light energy induces cell damage and increases the cost of microalgal cultivation. Daily zeaxanthin productivity should be considered in the context of reducing cultivation cost. Thus, the zeaxanthin productivity (mg·L^−1^·d^−1^) of *mp3* was calculated during the culturing period; low-light (7 days), mid-light (5 days), and high-light conditions (5 days) (Figure S3). Due to the reduced cultivation period, the daily zeaxanthin productivity of *mp3* under mid-light conditions was 0.400 mg·L^−1^, which was 36% higher than under the low-light conditions (0.295 mg·L^−1^). The daily productivity of the *zea1* mutant was also higher (by 20%) under mid-light conditions (0.373 mg·L^−1^) than under low-light conditions (0.310 mg·L^−1^). Thus, our results indicate that mid-light conditions are optimal for increasing zeaxanthin productivity.

In industry, a fed-batch cultivation method has been used extensively because of its diverse advantages [44]. One is the reduction of time needed to reach the stationary phase, which is the optimal time-point for compound production. The other is that it is easy to maintain the optimal nutrient composition of culture media and to regulate the cultivation process. Some reports have described the details of fed-batch cultivation for the production of valuable compounds [45,46,47]. Through simulation of the fed-batch cultivation process at the flask scale, we determined that the maximal zeaxanthin yield of the *mp3* mutant under optimized salinity and light conditions was 8 mg·L^−1^.

There have been many reports of zeaxanthin content in photosynthetic organisms, such as corn (*Zea mays* L.), wolfberry (*Lycium chinense*), marigold flower, and several Chlorophycean microalgae [8,48,49,50]. Among higher plants, zeaxanthin content is 0.0057 mg·g^−1^ in dried yellow dent corn, and 1 mg·g^−1^ (as zeaxanthin dipalmitate) in wolfberry. Marigold flowers, a well-known lutein source, contain only 5% of the total carotenoids of zeaxanthin (0.025 mg·g^−1^). Among freshwater microalgae, zeaxanthin content is 1 mg·g^−1^ in *Monoraphidium braunii*, 0.71 mg·g^−1^ in *Chlorella fusca*, and 0.46 mg·g^−1^ in *Scenedesmus vacuolatus*. Among genetically modified plants and algae, 0.04 mg·g^−1^ of zeaxanthin was reported in potato (*Solanum tuberosum* L.), and 2.4 mg·g^−1^ in *Chlamydomonas reinhardtii* [22,38,51]. In comparison with the above data, 6.6–7.0 mg·g^−1^ of zeaxanthin in the *mp3* mutant is a quite high level. Thus, this mutant has the potential to replace the present resources used for zeaxanthin production. Before commercialization, its zeaxanthin yield should be confirmed at an industrial scale, such as in a closed photo-bioreactor or an open raceway pond, because multiple variable factors may affect productivity in these settings. Thus, future studies should aim to determine the pattern of zeaxanthin production by zeaxanthin-accumulating mutants (*zea1*, *mp3*) during industrial-scale cultivation.

## 4. Materials and Methods

### 4.1. Strains and Culture Conditions

Wild type *Dunaliella* was isolated by Mordhay Avron [52] and was formerly named *Dunaliella salina*, but recent molecular phylogenetic characterization showed that it is very similar to *Dunaliella tertiolecta* [53]. Using genomic analysis, we confirmed the genomic similarity and renamed it *D. tertiolecta*. The *zea1* mutant strain, which cannot synthesize any β,β-epoxycarotenoids derived from zeaxanthin, was described previously [22]. It constitutively accumulates zeaxanthin, but lacks antheraxanthin, violaxanthin, and neoxanthin under all growth conditions.

The standard culture mode was a single-batch culture system with no additions or withdrawals during incubation. The growth medium was a slightly modified version of artificial saline water used by Pick et al. [54] and contained the following ingredients: 40 mM Tris–HCl (pH 7.4), 5 mM KNO_3_, 4.5 mM MgCl_2_, 0.5 mM MgSO_4_, 0.3 mM CaCl_2_, 0.1 mM K_2_HPO_4_, 2 μM FeCl_3_, 20 μM Na-EDTA, 50 μM H_3_BO_3_, 10 μM MnCl_2_, 0.8 μM ZnSO_4_, 0.4 μM CuSO_4_, 2 μM Na_2_MoO_4_, 1.5 μM NaVO_3_, 0.2 μM CoCl_2_, and 25 mM NaHCO_3_ as an inorganic carbon source. Both *D. tertiolecta* wild type and *zea1* were grown photoautotrophically, in 500 mL flasks, under 65–85 μmol photons·m^−2^·s^−1^ of continuous white fluorescent light, at 25 ± 1 °C. Cultures were shaken continuously on an orbital shaker (100 rpm). Second-generation EMS mutants were grown under the same conditions. During screening, all *Dunaliella* strains cultured in the medium were supplemented with 1.5 M NaCl. For salinity optimization of the culture medium, they were cultured in media containing 0.3 M, 0.6 M, 1.5 M, and 3.0 M NaCl, respectively. Experiments of light condition optimization and fed-batch culture were conducted at the optimized salinity, 0.6 M NaCl.

### 4.2. Mutagenesis and Mutant Selection

*zea1* cells were harvested in the exponential phase by centrifugation at 840× *g* for 5 min. The pelleted cells were re-suspended in the growth medium at a density of 2 × 10^8^ cells·mL^−1^. The EMS treatment method used was as published previously [22]. Two weeks after spreading on a solid agar medium, colonies that were brighter than *zea1* were selected for further analysis of pigment composition.

### 4.3. Experimental Culture Conditions

#### 4.3.1. Culture Medium Optimization: NaCl Concentration

To establish the effects of salinity on growth and xanthophyll pigment production, different NaCl concentrations (0.3 M, 0.6 M, 1.5 M, and 3.0 M) were assayed in 200 mL single-batch cultures in 500 mL culture flasks. The cells, after adapting to different salinities for 1 week, were inoculated at about 2.5 × 10^5^ cells·mL^−1^, and were cultured in low-light conditions (65–85 μmol photons·m^−2^·s^−1^), with orbital shaking (100 rpm). All experiments were conducted in triplicate.

#### 4.3.2. Culture Condition Optimization: Irradiance

To establish the effects of light intensity on growth and xanthophyll pigment production, three different irradiances were assayed in 200 mL single-batch cultures in 500 mL culture flasks: low-light (65–85 μmol photons·m^−2^·s^−1^), mid-light (140–160 μmol photons·m^−2^·s^−1^), and high-light (580–620 μmol photons·m^−2^·s^−1^). The cells, adapted to different light conditions for 1 week, were inoculated at about 2.5 × 10^5^ cells·mL^−1^, and were cultured by orbital shaking (100 rpm) on the medium containing 0.6 M NaCl, which was selected for culture medium optimization. All experiments were conducted in more than triplicate.

#### 4.3.3. Repeated Fed-Batch Culture

To measure zeaxanthin yield at maximal cell density in flask cultures, the repeated fed-batch culture system was used in pulse-feeding mode with multiple cycles. Cells were adapted to the medium containing 0.6 M NaCl. Thereafter, they were inoculated at about 2.5 × 10^5^ cells·mL^−1^ in 200 mL of culture medium in 500 mL culture flasks, and were cultured under mid-light conditions (140–160 μmol photons·m^−2^·s^−1^), with orbital shaking (100 rpm). When the cells reached the stationary phase, they were harvested by centrifugation at 840× *g* for 15 min and the culture medium was removed. Fresh culture medium was then added to the flask, and a new cultivation cycle was started. These processes were repeated until the cell density no longer increased. All experiments were conducted in triplicate.

### 4.4. Analytical Methods

#### 4.4.1. Specific Growth Rate and Biomass

Specific growth rate (μ) was calculated from the measured cell density, during the exponential phase, using the equation: μ = ln (D_2_/D_1_)/(t_2_ − t_1_), where D_2_ and D_1_ represent cell density (×10^4^ cells·mL^−1^) at times t_2_ and t_1_, respectively [55].

Microalgal biomass was measured as dry weight as described previously [22], with slight modifications: 100 mL of cell culture was harvested by centrifugation at 1500× *g* for 5 min at 20 °C. To remove cell-debris, the supernatant (medium) was carefully discarded, and the pellets were washed once. Cells were re-suspended and transferred to a pre-weighed 15 mL Falcon tube (A). Cells were harvested by centrifugation at 1500× *g* for 5 min at 20 °C, and the supernatant was completely removed. After drying for 24 h at 65 °C, the tubes were re-weighed (B). Net dry weight was calculated as “(B) − (A)”. Biomass was also quantified as cell density by direct cell counting using a hemocytometer (Marienfeld-Superior, Lauda-Königshofen, Germany) in the 25 central squares of the chamber.

#### 4.4.2. Pigment Analysis

For pigment extraction, samples were collected early in the stationary phase. Each 0.5 mL aliquot of cell culture (2.5 × 10^6^ cells·mL^−1^) was harvested by centrifugation (20,000× *g*, 2 min), and the supernatant was discarded. Pigments were extracted by pipetting the pellet into 0.5 mL of 90% (*w*/*w*) acetone for 1 min. After centrifugation at 20,000× *g* for 5 min, the supernatant was filtered through a 0.2-μm nylon filter. Filtrate was analyzed on a Shimadzu Prominence high-performance liquid chromatography (HPLC) system (model LC-20AD) (Shimadzu, Kyoto, Japan), equipped with a Waters Spherisorb S5 ODS2 cartridge column (4.6 × 250 mm) (Waters, MA, USA). HPLC analysis was performed according to the method described by Park et al. [56]. Pigments were identified by retention time and absorption spectra with reference to pigment standards (DHI 14C Centralen; Denmark). Concentrations of individual pigments were determined by HPLC using the same standards. To calculate daily zeaxanthin productivity, the content was divided by the culturing period.

## 5. Conclusions

The green alga *Dunaliella tertiolecta* can adapt to diverse saline environments and we consider it an ideal organism for carotenoid pigment production using natural seawater. The strain characterized in this study, *D. tertiolecta* mutant *mp3*, accumulated zeaxanthin under all growth conditions. In lab-scale culture, 0.6 M salinity and light intensity of 140–160 μmol photons·m^−2^·s^−1^ were selected as optimal conditions for zeaxanthin production. The maximal yield of zeaxanthin was 8 mg·L^−1^ under these conditions. Our results demonstrate that *D. tertiolecta mp3* is an interesting and promising marine microalga for zeaxanthin production and commercial applications.

## Figures and Tables

**Table 1:** Effect of NaCl concentration on cellular carotenoid content.

Strains	[NaCl]	Carotenoid Pigments (μg·10^6^·Cells^−1^)					
		Neoxanthin	Violaxanthin	Antheraxanthin	Lutein	Zeaxanthin	*β*-Carotene
wild type	**0.3 M**	0.112 (±0.008)	0.157 (±0.006)	0.061 (±0.005)	0.507 (±0.022)	0.079 (±0.005)	0.172 (±0.011)
	**0.6 M**	0.101 (±0.018)	0.112 (±0.014)	0.061 (±0.007)	0.414 (±0.012)	0.083 (±0.007)	0.133 (±0.013)
	**1.5 M**	0.096 (±0.018)	0.074 (±0.005)	0.057 (±0.003)	0.505 (±0.059)	0.117 (±0.013)	0.143 (±0.010)
	**3.0 M**	0.122 (±0.010)	0.086 (±0.004)	0.074 (±0.004)	0.433 (±0.027)	0.131 (±0.006)	0.149 (±0.013)
*zea1*	**0.3 M**	N.D.	N.D.	N.D.	0.358 (±0.017)	0.246 (±0.011)	0.186 (±0.008)
	**0.6 M**	N.D.	N.D.	N.D.	0.459 (±0.025)	0.303 (±0.038)	0.184 (±0.004)
	**1.5 M**	N.D.	N.D.	N.D.	0.502 (±0.058)	0.374 (±0.012)	0.185 (±0.010)
	**3.0 M**	N.D.	N.D.	N.D.	0.402 (±0.025)	0.359 (±0.038)	0.167 (±0.010)
*mp3*	**0.3 M**	N.D.	N.D.	N.D.	0.272 (±0.014)	0.262 (±0.019)	0.130 (±0.001)
	**0.6 M**	N.D.	N.D.	N.D.	0.329 (±0.016)	0.368 (±0.013)	0.171 (±0.015)
	**1.5 M**	N.D.	N.D.	N.D.	0.346 (±0.035)	0.389 (±0.012)	0.173 (±0.008)
	**3.0 M**	N.D.	N.D.	N.D.	0.268 (±0.024)	0.312 (±0.029)	0.133 (±0.013)

* Note: N.D. means “peak was not detected”.

**Table 2:** Effect of light intensity on cellular carotenoid content.

Strains	Irradiance	Carotenoid Pigments (μg·10^6^·Cells^−1^)					
		Neoxanthin	Violaxanthin	Antheraxanthin	Lutein	Zeaxanthin	*β*-Carotene
wild type	**Low-light**	0.101 (±0.018)	0.112 (±0.014)	0.061 (±0.007)	0.414 (±0.012)	0.083 (±0.007)	0.133 (±0.013)
	**Mid-light**	0.069 (±0.005)	0.091 (±0.003)	0.056 (±0.004)	0.324 (±0.023)	0.066 (±0.006)	0.119 (±0.006)
	**High-light**	0.051 (±0.002)	0.072 (±0.004)	0.041 (±0.002)	0.194 (±0.016)	0.043 (±0.001)	0.088 (±0.003)
*zea1*	**Low-light**	N.D.	N.D.	N.D.	0.459 (±0.025)	0.303 (±0.038)	0.184 (±0.004)
	**Mid-light**	N.D.	N.D.	N.D.	0.336 (±0.016)	0.253 (±0.012)	0.159 (±0.013)
	**High-light**	N.D.	N.D.	N.D.	0.236 (±0.006)	0.149 (±0.004)	0.079 (±0.003)
*mp3*	**Low-light**	N.D.	N.D.	N.D.	0.329 (±0.016)	0.368 (±0.013)	0.171 (±0.015)
	**Mid-light**	N.D.	N.D.	N.D.	0.240 (±0.003)	0.267 (±0.005)	0.174 (±0.023)
	**High-light**	N.D.	N.D.	N.D.	0.173 (±0.005)	0.209 (±0.002)	0.080 (±0.014)

* Note: N.D. means “peak was not detected”.

## Supplementary Materials

The following are available online at www.mdpi.com/1660-3397/15/6/189/s1, Figure S1: Zeaxanthin content and growth pattern of *D. tertiolecta* strains selected during mutant screening. (A) Zeaxanthin contents of wild type, *zea1*, and three mutants (based on chlorophyll *a*). The *mut12* was named as *mp3* mutant; (B) Growth pattern of wild type, *zea1*, and three mutants, Figure S2: Microscopic images of wild type *D. tertiolecta*, *zea1* mutant and *mp3* mutant. The plastid regions (green) of the wild type and *zea1* were larger than that of the *mp3* mutant strain, whereas auto-fluorescence signal patterns were similar. Left side, differential interference contrast (DIC) images; right side, auto-fluorescence (AF) images, Figure S3: Zeaxanthin productivity of wild type *D. tertiolecta*, *zea1* mutant and *mp3* mutant. Experimental light conditions were low-light (LL), mid-light (ML) and high-light (HL). Statistical analyses were performed using Student’s *t*-test, * *p* < 0.05. All experiments were conducted in more than triplicate, Table S1: Effects of salinity on volumetric and cellular biomass. (A) Volumetric biomass of wild type (WT), *zea1* and *mp3* mutant (g·L^−1^); (B) Cellular biomass of wild type (WT), *zea1* and mp3 mutant (mg·10^6^·cells^−1^), Table S2: Effects of irradiance on specific growth rates of wild type (WT), *zea1* and *mp3*.
