# Supplementary material for: Development of a Dunaliella tertiolecta Strain with Increased Zeaxanthin Content Using Random Mutagenesis
Source: Mar Drugs. 2017 Jun 21;15(6):189. doi: 10.3390/md15060189 (PMC5484139; doi:10.3390/md15060189)
Supplement: Supplementary file 1 [file marinedrugs-15-00189-s001.pdf]

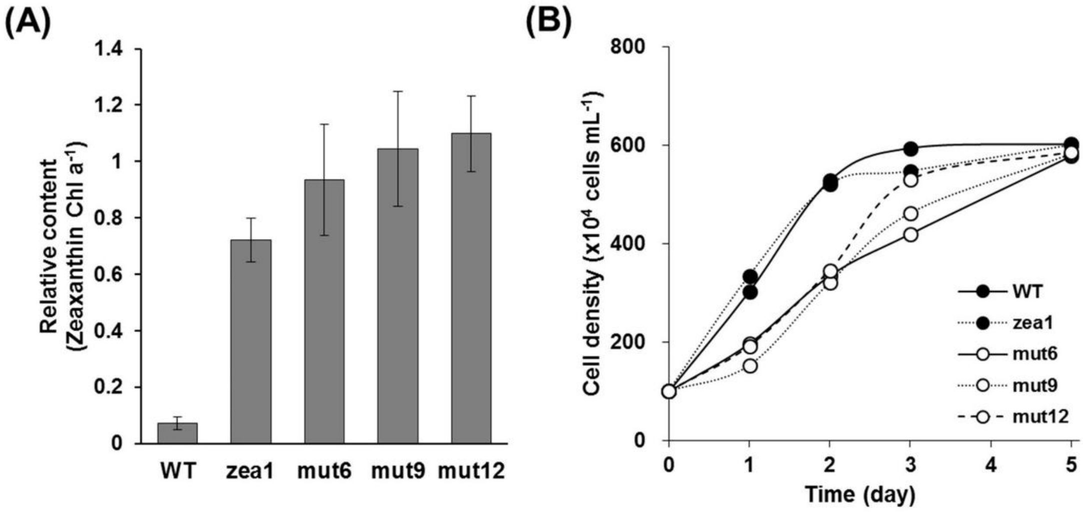

Figure S1. Zeaxanthin content and growth pattern of *D. tertiolecta* strains selected during mutant screening. (A) Zeaxanthin contents of wild type, *zea1*, and three mutants (based on chlorophyll *a*). The *mut12* was named as *mp3* mutant; (B) Growth pattern of wild type, *zea1*, and three mutants.

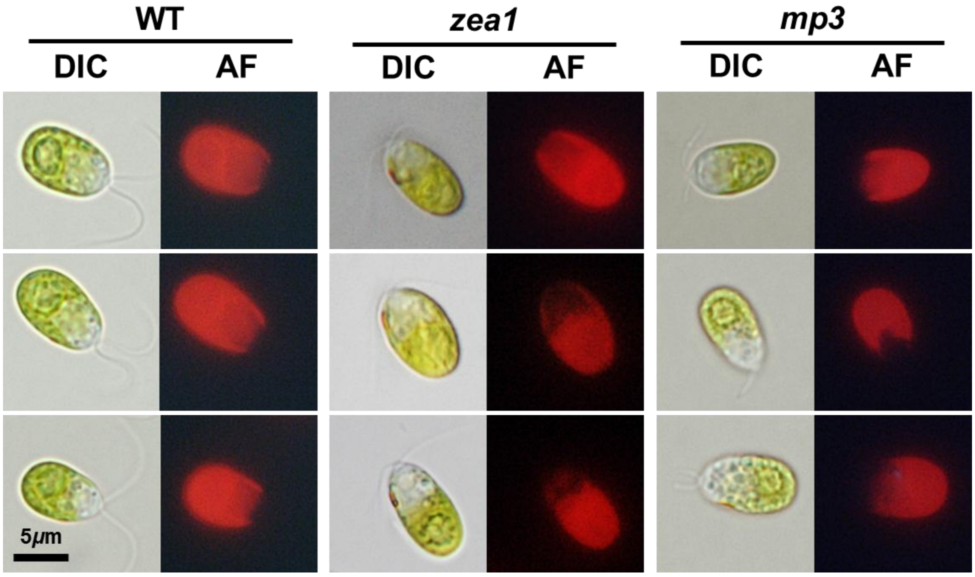

Figure S2. Microscopic images of wild type *D. tertiolecta*, and *zea1* and *mp3* mutants. The plastid regions (green) of the wild type and *zea1* were larger than that of the *mp3* mutant, whereas auto-fluorescence signal patterns were similar. Left side, differential interference contrast (DIC) images; right side, auto-fluorescence (AF) images.

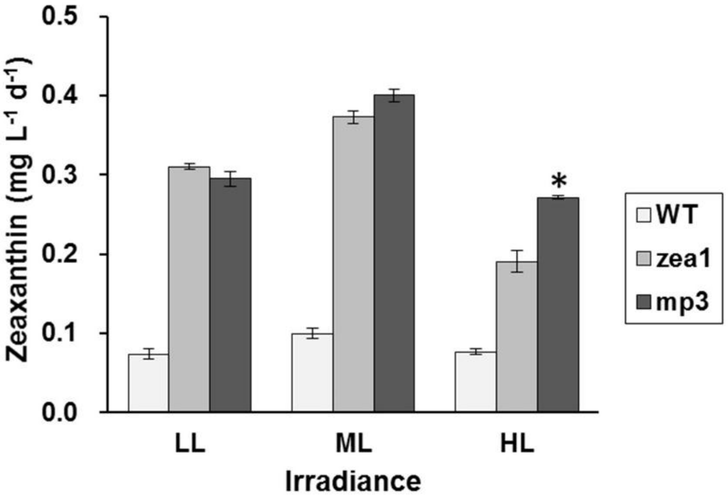

Figure S3. Zeaxanthin productivity of wild type *D. tertiolecta*, and *zea1* and *mp3* mutants. Experimental light conditions were low-light (LL), mid-light (ML) and high-light (HL). Statistical analyses were performed using Student's t-test, $^{*}p < 0.05$. All experiments were conducted in more than triplicate.

Table S1. Effects of salinity on volumetric and cellular biomass. (A) Volumetric biomass of wild type (WT), *zea1* and *mp3* mutant ($g \cdot L^{-1}$); (B) Cellular biomass of wild type (WT), and *zea1* and *mp3* mutants ($mg \cdot 10^6 \cdot cells^{-1}$).

(A)

$g \cdot L^{-1}$	DW (culture volume base)			
	**0.3 M**	**0.6 M**	**1.5 M**	**3.0 M**
**WT**	0.469 (±0.016)	0.458 (±0.011)	0.360 (±0.003)	0.259 (±0.010)
zea1	0.420 (±0.012)	0.420 (±0.009)	0.357 (±0.004)	0.239 (±0.015)
mp3	0.380 (±0.003)	0.364 (±0.005)	0.353 (±0.003)	0.245 (±0.010)

(B)

$mg \cdot 10^6 \cdot Cells^{-1}$	DW (cell number base)			
	**0.3 M**	**0.6 M**	**1.5 M**	**3.0 M**
**WT**	0.072 (±0.002)	0.069 (±0.001)	0.070 (±0.003)	0.060 (±0.003)
zea1	0.073 (±0.001)	0.068 (±0.002)	0.074 (±0.000)	0.065 (±0.002)
mp3	0.066 (±0.002)	0.065 (±0.002)	0.069 (±0.001)	0.064 (±0.004)

Table S2. Effects of irradiance on specific growth rates of wild type (WT), *zea1* and *mp3*.

d^{-1}	Specific growth rate (μ)		
	**Low-light**	**Mid-light**	**High-light**
**WT**	0.72 (±0.03)	0.97 (±0.01)	1.01 (±0.02)
zea1	0.54 (±0.02)	1.11 (±0.02)	1.03 (±0.03)
mp3	0.46 (±0.04)	1.18 (±0.02)	1.21 (±0.04)
